# Effect of Dexamethasone Implant on Subfoveal Choroidal Thickness in Early Period in Vitrectomized Eyes with Diabetic Macular Edema

**DOI:** 10.1155/2021/8840689

**Published:** 2021-04-12

**Authors:** A. Altun, A. M. Hacimustafaoglu

**Affiliations:** ^1^Bahcesehir University, Department of Ophthalmology, Istanbul, Turkey; ^2^VM Medical Park Goztepe Hospital, Clinic of Ophthalmology, Istanbul, Turkey

## Abstract

**Aim:**

To investigate the change in subfoveal choroidal thickness (SFCT) in vitrectomized eyes with intravitreal dexamethasone (IVD) implant injection for the treatment of diabetic macular edema (DME).

**Method:**

In this prospective and controlled study, the vitrectomized eyes of diabetic patients were included. The study group (Group 1) was formed by diabetic vitrectomized eyes with DME. The control group (Group 2) was formed by diabetic vitrectomized eyes without DME. Only one intravitreal IVD implant was injected into the eyes in Group 1. In the first, second, and fourth months, choroidal layers were measured by optical coherence tomography and complete ophthalmologic examinations were performed for all cases.

**Results:**

Ninety-six eyes of 96 cases were included in the study. There were 48 eyes of 48 different patients in each group. After IVD injection, statistically significant improvement was observed in the best corrected visual acuity in Group 1. The mean SFCT in eyes with DME was statistically significantly thinner (*p* < 0.01) and thinness became more pronounced during the four-month follow-up period after IVD implant injection (*p* < 0.01).

**Conclusion:**

In the presence of DME in vitrectomized eyes, the thinness of the SFCT may become evident after dexamethasone implant injection.

## 1. Introduction

Diabetes mellitus (DM) is a disease that often causes pathology in the vascular network in the body [1]. Diabetic macular edema (DME) is one of the most important causes of vision loss in diabetic patients [2]. Many approaches have been recently conducted to prevent or treat DME. Strict metabolic control, argon laser photocoagulation, and vitrectomy are some of the main options [3]. Anti-VEGF drugs that are used in several macular diseases are also an important treatment option for DME [4]. The effective results of intravitreal dexamethasone (IVD) implant in the treatment of DME suggest that inflammation may also play an important role in the pathogenesis [5–7].

Studies examining the effects of DM and DME on the choroid have been recently conducted. In this study, we investigated the effect of IVD implant, which is used for the treatment of DME, on subfoveal choroidal thickness (SFCT) in diabetic eyes that previously had pars plana vitrectomy (PPV).

## 2. Methods

In this prospective and controlled study, vitrectomized eyes of the diabetic patients that were presented to our clinic between January 2019 and December 2019 were included. The study group (Group 1) and the control group (Group 2) were formed by the eyes with DME and without DME, respectively. Grouping was done randomly. Only one intravitreal IVD implant injection was applied to the eyes in Group 1. Approval was obtained from the University of Health Sciences Ethics Committee, Istanbul, Turkey. Informed consent was obtained from all patients, and the study was conducted in accordance with the principles of the Declaration of Helsinki.

Patients with uncontrolled glaucoma, corneal or optic nerve pathology, amblyopia, cataract, uveitis, hemoglobin A1c greater than 10%, and history of laser application to the macula were excluded from the study. Only one eye of each patient was included in the study. A randomized selection was made for patients with the features of including both eyes.

During the four-month follow-up period, SFCT was measured by spectral-domain optical coherence tomography (OCT) with enhanced depth imaging technique, and complete ophthalmological examination was made monthly. In addition, fundus fluorescein angiography (FFA) imaging of all eyes was performed before the study and at the fourth month. Retinal images were followed by the same FFA and OCT device (Topcon 3D OCT‐2000, Tokyo, Japan). All findings were evaluated by the same retina specialist (AA). The imaging technician and assessor were masked. The best corrected visual acuity (BCVA) was measured with the Snellen chart and converted to logMAR equivalents for statistical analysis. The obtained data were compared with each other.

The distribution of variables was measured by the Kolmogorov–Smirnov test. Mann–Whitney *U* test was used to analyze quantitative independent data. Chi-square test was used in the analysis of qualitative independent data. Wilcoxon test was used in the analysis of dependent quantitative data. Statistical significance level was set at *p* < 0.05. SPSS 26.0 program was used in the analyzes.

## 3. Results

A total of 96 eyes of 96 cases were included in this study. In both groups, there were 48 eyes of 48 patients. The mean age of Group 1 and Group 2 was 54.2 ± 4.8 and 53.8 ± 4.7 years, respectively. The male : female ratio in Group 1 and Group 2 was 20 : 28 and 23 : 25, respectively. The number of cases with arterial hypertension was 28 (58.3%) and 26 (54.1%) in Group 1 and Group 2, respectively. The number of the patients taking anticoagulant medication was 16 (33.3%) and 14 (29.1%) in Group 1 and Group 2, respectively. The number of the patients taking antiplatelet medication was 16 24 (50.0%) and 19 (39.5%) in Group 1 and Group 2, respectively. The mean hemoglobin A_1C_ in Group 1 and Group 2 was 7.67 ± 0.35% and 7.41 ± 0.33%, respectively. There was no statistically significant difference between the groups in terms of age, gender, hemoglobin A_1C_ level, and frequency of anticoagulant or antiplatelet drug use (*p* > 0.05). In Group 1 and Group 2, the mean total number of anti-VEGF injection that applied to the eyes before the study was 3.83 ± 0.78 and 1.97 ± 0.42, respectively. The mean number of anti-VEGF injection was statistically significantly higher in Group 1 than Group 2 (*p* < 0.01) (Table 1).

Before the study, BVCA in Group 1 and Group 2 were 0.62 ± 0.17 and 0.11 ± 0.03 logMAR, respectively. The mean BCVA in Group 2 was statistically significantly higher than Group 1 before the study (*p* < 0.01). In Group 1, the mean BCVA in the follow-up period after IVD implant injection was 0.32 ± 0.14, 0.12 ± 0.10, and 0.11 ± 0.06 logMAR at the first, second, and fourth months, respectively. The mean BCVA in Group 1 improved after IVD implant injection at first and second months (*p* < 0.01). There was no statistically significant change in mean BCVA in Group 2 during the four-month follow-up period (*p* > 0.05). The mean BCVA in Group 1 improved significantly at the first month after IVD implant injection (*p* < 0.01). There was no statistically significant difference between the groups in terms of mean BCVA levels at the second and fourth months (*p* > 0.05) (Table 2).

The mean SFCT before IVD implant injection was 205.8 ± 8.2 and 295.0 ± 4.6 *μ*m in Group 1 and Group 2, respectively. The mean SFCT in Group 1 was statistically significantly thinner than Group 2 (*p* < 0.01) before the injection. In Group 1, the mean SFCT during the follow-up period was 193.8 ± 7.2, 191.3 ± 6.3, and 187.4 ± 6.6 *μ*m in the first, second, and fourth months, respectively. In Group 1, after IVD implant injection, the mean SFCT decreased in the follow-up period when compared to preinjection (*p* < 0.05). In Group 2, the mean SFCT during the follow-up period was 292.3 ± 4.9, 295.4 ± 4.1, and 297.7 ± 4.5 *μ*m in the first, second, and fourth months, respectively. There was no significant change in the mean SFCT in Group 2 within the follow-up period (*p* > 0.05) (Table 3).

## 4. Discussion

The choroid is the nutrient source of the outer retina and its structure consists of blood vessels. It provides thermoregulation, affects the retina due to changes in its thickness, and regulates the secretion of various mediators [8]. These mediators could be the vascular endothelial growth factor (VEGF) and proinflammatory cytokines that may also play important role in the pathogenesis of DME [9].

There are studies in the literature that have been conducted to investigate the effect of DME on choroidal thickness [10–13]. Eliva et al. investigated the effect of DME on SFCT in a prospective study on 106 eyes and reported that the main choroidal thickness was significantly thinner in eyes with DME than in healthy eyes [10]. Lains et al. investigated the effect of diabetic retinopathy (DR) on SFCT in their multicenter and prospective study and reported that SFCT thickness in eyes with DR was statistically significantly thinner than in healthy eyes [11]. Rewbury et al. investigated the effect of DR and DME on SFCT in a retrospective study and reported that in nonproliferative cases as the DR grade worsens there was a decrease in SFCT thickness, but a significant increase in proliferative cases [12]. Kniggendorf et al. retrospectively investigated the change in SFCT in eyes with intravitreal anti-VEGF injection for DME treatment and reported that significant thinning developed in SFCT after anti-VEGF injection during the six-month follow-up period [13]. In a prospective study, Okamoto et al. investigated the change in SFCT after intravitreal ranibizumab injection on 28 eyes with DME and reported that after ranibizumab injection, a significant decrease in choroidal perfusion, vascular index, and thickness developed [14]. In our study, the mean SFCT in vitrectomized eyes with DME was statistically significantly thinner than the eyes without DME. The reason for the thinning in SFCT in our study group may be due to previous anti-VEGF drug injections. All these studies suggest that one of the most important factors in SFCT may be the perfusion of the choroid. In eyes with DR or DME, the injection of anti-VEGF drugs may decrease SFCT thickness due to regression of neovascularization or perfusion of the choroid. In our study, the higher mean number of anti-VEGF injections in the study group with DME supports this hypothesis.

IVD implant is widely used in the treatment of retinal vein occlusion, noninfectious uveitis, and DME [15]. In the presence of vitreomacular traction syndrome or refractory edema after vitrectomy, IVD implant may be a treatment option for retina specialists in the treatment of DME. In their international and multicenter study, Iglicki et al. investigated the efficacy of IVD implant in DME and reported that the improvement in visual acuity was more pronounced in naive eyes when compared with the eyes refractory to anti-VEGF drugs [16]. In another study, Iglicki et al. investigated the long-term effect of the IVD implant on the severity and progression of nonproliferative DR and reported that both the progression and the severity of DR and proliferative DR decreased in eyes which received dexamethasone injection [17]. In a comparative and retrospective study, it was reported that IVD implant injection with silicone oil tamponade during diabetic tractional retinal detachment surgery significantly improved the severity of proliferative vitreoretinopathy and reduced the risk of redetachment [18].

Studies investigating biomarkers and predictive factors in DME have been conducted in the literature [19]. It has been reported that the presence of subretinal fluid, the absence of hyper-reflective foci, and integrity of the inner segment-outer segment layer could be OCT biomarkers for better functional success in eyes that received IVD implant injection for DME treatment [20]. In their multicenter and retrospective study, Zur et al. reported for the first time in the literature that the IVD implant could ameliorate disorganization of retinal inner layers which is a biomarker in eyes with DME [21].

In a prospective study, Mathis et al. reported that the increase in choroidal thickness in eyes with DME is an indicator for exudative recurrence that regressed with anti-VEGF or IVD implant injection [22]. Kim et al. investigated the effect of IVD implant on eyes with DME resistant to anti-VEGF drugs and reported that IVD implant causes an increase in BCVA and a decrease in the central fovea and SFCT thickness [23]. Lee et al. reported that IVD implant may cause thinning not only in eyes with DME but also in eyes with retinal vein occlusion [24]. In the pathogenesis of the active phase of DME, not only VEGF but also proinflammatory cytokines may have a role. Because most of these mediators could cause vasodilatation and increase choroidal thickness [25]. In our study, a statistically significant thinning developed in the mean SFCT in the follow-up period after IVD implant injection in vitrectomized diabetic eyes with DME. These results may occur due to the decrease in vasodilator effect of proinflammatory cytokines and mediators.

Small sample and short follow-up period are the limitations of this study. More comprehensive long-term randomized clinical studies are needed to investigate the effect of dexamethasone implant on choroidal thickness.

## 5. Conclusion

Choroidal thickness is thinner in vitrectomized eyes with diabetic macular edema. Dexamethasone intravitreal implant may be an effective treatment option for diabetic macular edema and cause thinning in subfoveal choroidal thickness. Choroidal thickness could be an important biomarker for diabetic macular edema in vitrectomized eyes.

## Figures and Tables

**Table 1:** Demographic characteristics of the groups, hypertension history, anticoagulant and antiplatelet drug use, hemoglobin A1c levels, and previous anti-VEGF injections.

		Group 1 (with DME)					Group 2 (without DME)			*p* value	
		Mean ± SD/*n*−%			Median	Mean ± SD/*n*−%			Median		
Age (year)		54.2	±	4.8	55.0	53.8	±	4.7	54.0	0.872	^m^
Gender	Male	20		41.7%		23		47.9%		0.751	^X²^
	Female	28		58.3%		25		52.1%			

Hypertension		28		58.3%		26		54.1%		0.889	^X²^
Anticoagulant drug use		16		33.3%		14		29.1%		0.712	^X²^
Antiplatelet drug use		24		50.0%		19		39.5%		0.442	^X²^
Hemoglobin A1c (%)		7.67	±	0.35	7.75	7.41	±	0.33	7.61	0.714	^m^

Total number of anti-VEGF injections		3.83	±	0.78	4.00	1.97	±	0.42	2.00	0.000	^m^

^m^Mann–Whitney *U* test; ^X²^chi-square test; ^w^Wilcoxon test; SD, standard deviation; HbA1c, hemoglobin A1c; DME, diabetic macular edema; VEGF, vascular endothelial growth factor.

**Table 2:** Change in best corrected visual acuity of the groups.

	Group 1 (with DME)		Group 2 (without DME)		*p* value	
	Mean ± SD	Median	Mean ± SD	Median		
BCVA (logMAR)						
Preinjection	0.62 ± 0.17	0.69	0.11 ± 0.03	0.09	0.000	^m^
Postinjection 1^st^ month	0.32 ± 0.14	0.39	0.11 ± 0.03	0.09	0.000	^m^
Postinjection 2^nd^ month	0.12 ± 0.10	0.15	0.10 ± 0.03	0.09	0.312	^m^
Postinjection 4^th^ month	0.11 ± 0.06	0.09	0.10 ± 0.02	0.09	0.763	^m^

^m^Mann–Whitney *U* test; SD, standard deviation; BCVA, best corrected visual acuity; DME, diabetic macular edema.

**Table 3:** Change in subfoveal choroidal thickness of the groups.

	Group 1 (with DME)		Group 2 (without DME)		*p* value	
	Mean ± SD/*n*−%	Median	Mean ± SD/*n*−%	Median		
SFCT (*μ*m)						
Preinjection	205.8 ± 8.2	207.0	295.0 ± 4.6	294.0	0.000	^m^
Postinjection 1^st^ month	193.8 ± 7.2	195.0	292.3 ± 4.9	293.0	0.000	^m^
Postinjection 2^nd^ month	191.3 ± 6.3	193.0	295.4 ± 4.1	295.0	0.000	^m^
Postinjection 4^th^ month	187.4 ± 6.6	190.5	297.7 ± 4.5	296.0	0.000	^m^

^m^Mann–Whitney *U* test; SFCT, subfoveal choroidal thickness; SD, standard deviation; *μ*m, micrometer; DME, diabetic macular edema.

## Data Availability

The data analyzed during the current study are not publicly available due to the limitation of the hospital's archive system, but can be obtained from the corresponding author upon reasonable request.
